# Umbilical catheter placement aided by coronary guidewires

**DOI:** 10.1186/s40348-023-00155-5

**Published:** 2023-03-14

**Authors:** Katarzyna Gendera, Stanimir Georgiev, Peter Ewert, Stefan Eckstein, Christoph Fusch, Niels Rochow

**Affiliations:** 1 Department of Pediatric Cardiology and Congenital Heart Defects, German Heart Center Munich, Technische Universität München, Munich, Germany; 2 Eckstein Design, Munich, Germany; 3 Department of Pediatrics, Paracelsus Medical University, Breslauer Str. 201, 90471 Nuremberg, Germany; 4 Department of Pediatrics, McMaster University, Hamilton, Ontario Canada; 5 Department of Pediatrics, University Medical Center Rostock, Rostock, Germany

**Keywords:** Umbilical vessels, Coronary guidewire, Neonatology

## Abstract

Catheterization of the umbilical vessels has proven to be an effective and relatively rapid method for gaining central vascular access in neonates. However, it can be technically difficult, the procedure may last 30 min or longer, and it can be associated with complications in some patients. We suggest using a coronary guidewire during catheterization of umbilical vessels to support the placement of umbilical catheters and significantly reduce a risk for complications. We tested the proposed technique in 6 successful ex vivo bench tests of catheterization of the umbilical vessels in stillborn piglets immediately after birth. We are confident that using coronary guidewire as a guiding tool during catheterization of the umbilical vessels is a rapid and safe method. We expect that it allows to obtain a vascular access with lower risk for dangerous procedural complications, which could be a lifesaving in critically ill patients. However, the approach needs to be validated in a comparative study in neonates.

## Main text

Catheterization of the umbilical vein and arteries has proven to be an effective and relatively rapid method for gaining central vascular access in neonates. However, catheterization of the umbilical vessels can be technically difficult, takes more than 30 min, and can be associated with vascular complications and misplaced catheters in some patients [1–4].

The presence of anatomical narrowing at the level of curved junction of umbilical arteries between the abdominal wall and the peritoneum following the umbilical stump increases the risk for false passage and dissection.

Furthermore, the U-turn-like trajectory of the umbilical artery between its origin from the iliac artery and the fundus of the urinary bladder represents another potential obstacle when aiming cannulation. Due to the stiffness of umbilical catheters, these tend to move forward in a straight-line direction.

Particularly, the outer wall of an empty urinary bladder does not have enough resistance to ensure the normal anatomical course of the umbilical arteries during mechanical manipulation. The vascular passage could kink or even perforate.

Finally, accidental/iatrogenic perforations of the internal iliac artery just below the bifurcation of the common iliac artery were described.

Originally developed for interventions inside coronary arteries, but currently also commonly employed in other percutaneous interventions, coronary guidewires are used to navigate within vessels with a high risk for injury throughout the body. Accordingly, coronary guidewires have flexible and soft tips to minimize the risk of vessel dissection or perforation.

We propose a modified procedure for umbilical vessel cannulation by using a coronary guidewire during catheterization of umbilical arteries and the umbilical vein to support the placement of umbilical catheters and significantly reduce vessel injuries.

**Fig. 1:**
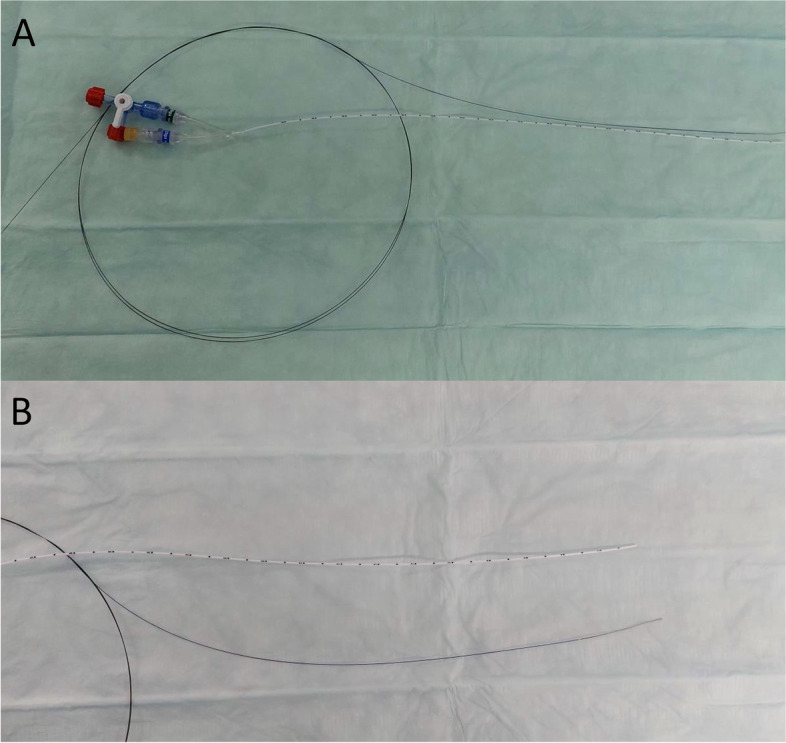
Coronary guidewire and umbilical catheter. **A** Guidewire loop and umbilical catheter with luer-lock connector. **B** Tip soft end of guidewire and umbilical catheter

We have tested the proposed technique in 6 successful ex vivo bench tests of catheterization of the umbilical arteries and umbilical veins in stillborn piglets immediately after birth. Abbott BMW Wire Hi-Torque Balance Middleweight Universal™ Guide Wire 0.014″, 190 cm (Abbott Laboratories, Illinois), and polyurethane umbilical catheter with a rounded tip (Umbili Cath^TM^, Utah Medical Products Inc., Midvale, Utah) were used (Fig. 1A,B). In all performed experiments, it was feasible to safely achieve a fast and secure vascular access through the umbilical vessels without perforation. Based on this experience, we suggest novel guided placement of the umbilical catheter.

**Fig. 2:**
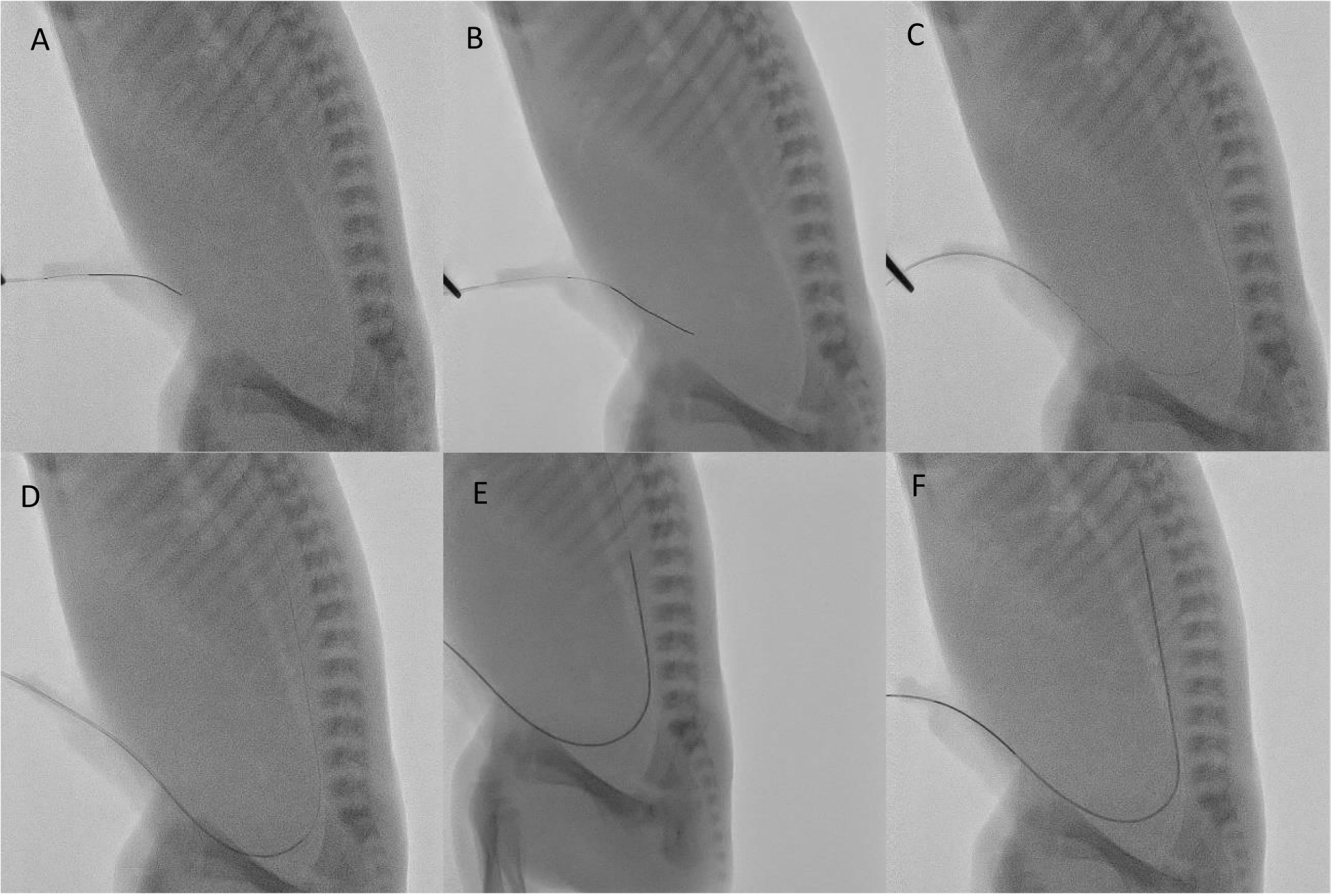
Placement of umbilical artery catheter (UAC). **A**, **B** Coronary guidewire (CGW) passing through the umbilicus. **C** CGW reaching the descending aorta and the umbilical catheter sliding through the umbilicus. **D**–**F** UAC passing over the CGW towards thoracic and descending aorta

The procedure of placing umbilical artery and umbilical vein catheters is modified from recent publications [5–7], and the following steps will be added and adjusted:

1. An umbilical catheter will be loaded with a coronary guidewire. The guidewire’s tip of the “soft” end will be kept inside the tip of the umbilical catheter. It should be noted that coronary guide wires have a length of up to 190 cm. For reasons of practicability, the “stiff” end could remain in the packaging or alternatively be shortened.
2. A fine dilator or fine forceps will be used to gently ease introducing the umbilical catheter into the dilated entrance of the vessel at the umbilical stump.
3. The position of the umbilical catheter inside the stump should be secured and the tip of the coronary wire ought to be gently pushed forward moving the tip distal out of the umbilical catheter. The soft tip of the guidewire will pass the umbilicus, move inferiorly inside the umbilical artery, then in the anterior division of the internal iliac artery, into the common iliac artery, and subsequently into the aorta (Fig. 2A–C).
4. Following the guidewire, the umbilical catheter will be pushed into the umbilical artery over the wire. The tip of the catheter should be placed ideally at high position at T6 to T10 level (Fig. 2C–F). The low position at the L3 to L5 level would be acceptable. The placement of the guidewire could be confirmed by ultrasound. Alternatively, the guidewire could be marked at a certain length, e.g., with a torque device, to prevent from deep insertion.
5. The guidewire will be removed and the catheter flushed with saline, e.g.
6. The position of the catheter should be confirmed using either ultrasound or x-ray.

The coronary guidewire-aided placement of umbilical catheter could be adopted for the umbilical vein. A similar technique could be applied while the guidewire and catheter would generally pass directly superiorly following the umbilical vein. The tip should be placed at the junction of the inferior vena cava with the right atrium. Coronary wire-aided placement for umbilical catheters allows us to gently pass the narrowed and angled segments of umbilical vessels. With this approach, it could be possible to avoid invasive alternative approaches for vascular access [8].

Indication for umbilical catheter placement has become restrictive in the recent time. As a result, safety aspects have become even more relevant. In conclusion, using coronary guidewire as a guiding tool and additional support during catheterization of the umbilical vessels is feasible. Successful, fast, and safe umbilical vessel access can be achieved by avoiding dangerous procedural complications, which could be a lifesaving in critically ill patients. However, future studies need to prove our results in clinical trials.

## Data Availability

All data supporting our findings were included in this manuscript.
